# Editorial: Gastric microbiota dysbiosis and gastric diseases

**DOI:** 10.3389/fmicb.2024.1390896

**Published:** 2024-04-25

**Authors:** Yan Tian, Ming Zhao, Xiangsheng Fu

**Affiliations:** Department of Gastroenterology, Clinical Medical College, The First Affiliated Hospital of Chengdu Medical College, Chengdu, Sichuan, China

**Keywords:** gastric microecology, gastric diseases, *H.pylori*, microbial dysbiosis, gastric microbiota transplantation

It has been estimated that trillions of microorganisms reside in the gastrointestinal tract, and play key roles in epithelial differentiation, immune system maturation, nutrient absorption, and metabolism (Belkaid and Hand, 2014). The diversity and abundance alterations are related to a variety of gastrointestinal diseases. The reshape of gastrointestinal microecology has become a research hotspot to treat gastrointestinal diseases. In this Research Topic, Gao et al. reported that moringa oleifera leaf relieved constipation by modulating the bacterial homeostasis, with the increasement of the abundance of microbiota that “treats constipation,” such as *Yricocus, Tyzzerella*, and *Desulfovibrio*, and the inhibition of some key “constipation causing” microbiota, such as *Bacteroidetes, Clostridium*, and *Bacteroidetes*. Du et al. reported that prebiotics can improve the structure of intestinal microbiota in children with functional diarrhea. Kudra et al. discussed the strengthening effect of postbiotics, including extracellular polysaccharides, cell wall fragments, tryptophan metabolites, enzymes, bacterial lysates, extracellular vesicles, and short-chain fatty acids, on gut microbiome and the potential application as new therapeutic options for anti-cancer treatment. Given that the gastrointestinal microecology is closely correlated to diseases, Li et al. conducted metagenomic sequencing of the microbial DNA in feces, and indicated the potential of the gut microbiome and the metabolites as non-invasive biomarkers of COPD.

The human stomach has an unusual gastric microecology, with much lower diversity and abundance of gastric microorganisms compared with that in intestine. In this Research Topic, Zhang et al. summarized the correlation between dysbiosis of gastric microecology and a variety of gastric diseases, including ulcers, functional gastric diseases and cancer, etc. A two-sample Mendelian randomization study showed a close relationship between gut microbiota and peptic ulcer disease, especially gastric ulcer and duodenal ulcer. Butyricococci and Peptococcus have harmful effects on GU, while Lachnospiraceae and Mollicutes have beneficial effects on GU (Dong et al.). Both Dong et al. and Zhang et al.'s articles indicated that *Helicobacter pylori* (*H.pylori*) was the most important pathogen leading to peptic ulcer disease and gastric cancer. However, the non-*H.pylori* associated dysbiosis of gastric microecology has been gradually recognized in recent years. Xu et al. reported that bile reflux was also considered an independent risk factor for gastric cancer, and deoxycholic acid could participate in the occurrence and development of gastric cancer by affecting the gastric microbiota. When it comes to gastric cancer, even *H.pylori* is eradicated or not infected, gastric cancer can still develop in gastric mucosa (Zhang et al.).

In addition to fecal microbiota transplantation, which has been demonstrated to be effective to treat intestinal diseases (Allegretti et al., 2019), gastric microbiota transplantation (GMT) is considered to reconstruct the gastric microecology for the treatment of gastric diseases. However, there is currently no relevant report of the method and safety of GMT. Therefore, clinical trials are needed to verify its clinical efficacy and safety.

## Author contributions

YT: Writing – original draft. MZ: Writing – review & editing. XF: Funding acquisition, Writing – review & editing.

## References

[B1] Allegretti J. R. Mullish B. H. Kelly C. Fischer M. (2019). The evolution of the use of faecal microbiota transplantation and emerging therapeutic indications. Lancet 394, 420–431. 10.1016/S0140-6736(19)31266-8 31379333

[B2] Belkaid Y. Hand T. W. (2014). Role of the microbiota in immunity and inflammation. Cell 157, 121–141. 10.1016/j.cell.2014.03.011 24679531 PMC4056765

